# Acute Upper Limb Ischemia and Amputation Post Antecubital Fossa Cannulation: A Report of Two Postpartum Patients

**DOI:** 10.7759/cureus.37575

**Published:** 2023-04-14

**Authors:** Vagisha Sharma, Rockey Dahiya, Mohd Junaid Ansar, Rishikesh Kumar, Chintamani Chintamani

**Affiliations:** 1 Medicine, Vardhman Mahavir Medical College and Safdarjung Hospital, Delhi, IND; 2 Medicine, Pandit Bhagwat Dayal Sharma Post Graduate Institute of Medical Sciences, Rohtak, IND; 3 General Surgery, Vardhman Mahavir Medical College and Safdarjung Hospital, Delhi, IND

**Keywords:** quality improvement and patient safety, ischemic gangrene, hypercoagulable state, iatrogenic complication, postpartum complication, hand amputation, arterial cannulation, intravenous cannulation

## Abstract

Upper extremity arterial thrombosis is less common than that in the lower extremity. Upper extremity arterial thrombosis, when present, is more likely to occur on the ulnar side of the circulation. Severe ischemia resulting from radial artery thrombosis is rare, but iatrogenic cannulation is the most common etiology when it occurs. The risk factors underlying this dreadful presentation are numerous and still under investigation. Pregnancy and the immediate postpartum period are physiological hypercoagulable states. Here we present unusual cases of acute limb ischemia post iatrogenic cannulation in two patients within six weeks postpartum. At four weeks postpartum, a 26-year-old para-1 live-1 female presented to the emergency department with swelling in her right upper limb for four weeks and its blackish discoloration for one week. A 24-year-old primigravida female who had a termination of a blighted ovum 12 days ago presented to the emergency department with gangrenous changes in her right hand and forearm. Both patients reported recent antecubital fossa cannulation within six weeks postpartum, triggering gangrenous hand changes. Both patients had to undergo amputation of the digits and hand ultimately. Thus we postulate the need for extra care and education of healthcare workers in the cannulation of pregnant and post-pregnancy patients to prevent limb-threatening complications.

## Introduction

Pregnancy is a physiological prothrombotic state. This is attributable to derangements seen in all components of Virchow's triad. The obstruction to venous flow from an enlarged gravid uterus and hormonally induced decreased venous tone contribute to Venous Stasis. Endothelial damage can result from venous hypertension or the consequence of delivery causing damage to pelvic veins. Hypercoagulability can be attributed to the increased levels of coagulation factors II, VII, VIII, X, and fibrin and decreased levels of anticoagulant factors like free protein S produced by the liver, and decreased fibrinolytic activity [1]. This is most commonly associated with complications related to venous thromboembolism [2]. Smoking, superficial vein thrombosis, and obstetric hemorrhage are some of the independent risk factors for venous thrombosis in pregnancy [3]. Acute limb ischemia (ALI) is a rare presentation in pregnancy and the postpartum period and presents as acute onset of limb pain, which progresses to paraesthesia, weakness, and paralysis [4]. Post-pregnancy ALI has been more often reported as a thromboembolic event than thrombosis [5]. Post-pregnancy arterial occlusion event is rarer than venous occlusion. Its occurrence has been associated with a history of obstetric complications or resulting from an iatrogenic injury sustained while managing these complications [5]. According to estimates, lower-extremity thrombosis is six times more common than upper-extremity thrombosis. The most frequent cause of upper extremity arterial thrombosis is repeated blunt trauma to the hypothenar eminence. This condition is more common on the ulnar side of the circulation. Radial artery thrombosis is rare, and when it does occur, it is usually due to iatrogenic cannulation, which results in thrombosis and emboli [6]. Here we present the case of two patients who presented with acute upper limb ischemia within six weeks postpartum and postabortion, respectively, and both reported a history of antecedent antecubital fossa cannulation.

## Case presentation

Case 1 

**Figure 1:**
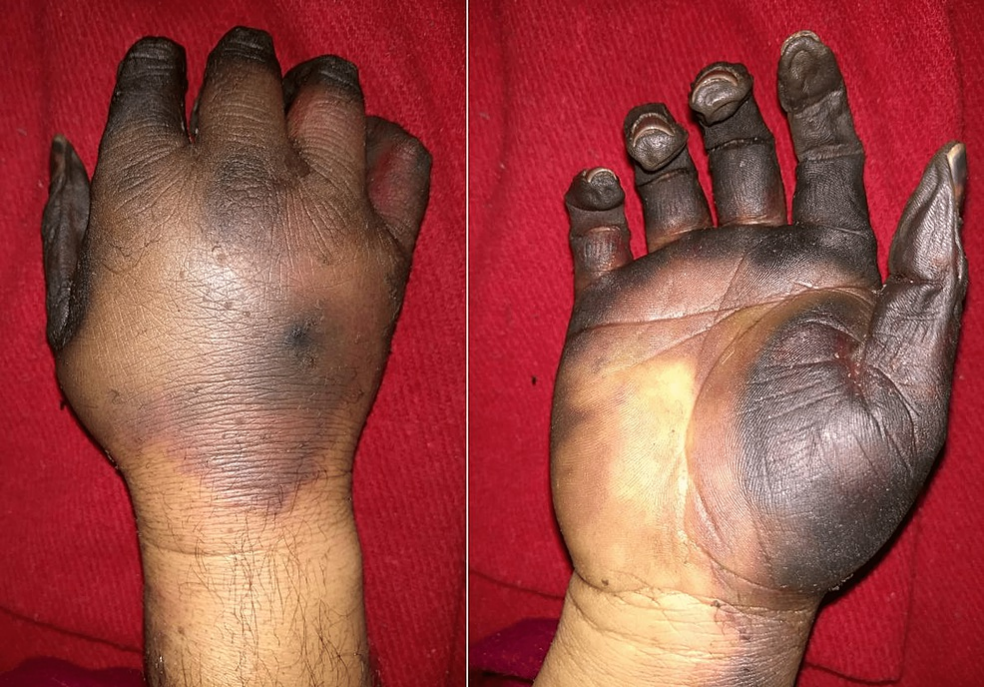
Dry gangrene of all fingers extending into the palm and dorsum of the hand.

A 26-year-old para-1 live-1 presented to the emergency department with swelling in her right upper limb for four weeks and its blackish discoloration for one week. She was well four weeks ago when she underwent antecubital fossa cannulation at a primary care hospital on her postpartum day two. On discharge, she started developing swelling in the right upper limb, which was gradually progressive. On examination in the emergency department after four weeks, brachial, radial, and ulnar pulsations were present but feeble compared to the left upper limb. The patient had no known comorbidity. She delivered a single healthy fetus four weeks ago. The patient was vitally stable and conscious, oriented to time, place, and person. Ultrasound arterial Doppler showed triphasic flow in the brachial artery and biphasic flow in proximal and distal radial and ulnar arteries. Ultrasound of venous Doppler was suggestive of superficial thrombophlebitis of the right cephalic vein and no evidence of deep vein thrombosis. Liver and kidney function tests and complete blood cell count were within normal limits. The patient underwent a release incision for compartment syndrome in her hand. Then, within one week, the patient developed blackish discoloration of digits which progressed to all digits and the dorsum of the hand (Figure 1). Surgery was planned after a definitive line of demarcation developed around the gangrenous portion of the hand. The patient was put on antibiotics IV amoxiclav 1.2 gm TDS and anticoagulant tab warfarin 2 mg OD.

Additionally, tab cilostazol 100 mg BD was given to increase the blood flow. An operation was done to disarticulate right-hand fingers at proximal interphalangeal joints with serial debridement and dressing. Intraoperatively proximal interphalangeal joints were identified, and ligaments were cut. Joint space was visualized, bleeders sutured, and compression packing was done. The release incision was not closed as it was left to heal by secondary intention because of underlying edema and slight muscle necrosis; these were managed by serial debridement and dressings. Her post-operative condition was stable, with the only complaint being sinus tachycardia, for which metoprolol was started after a cardiology consultation. Antibiotics were continued till post-op day five. The patient was discharged on anticoagulants and cilostazol with the advice to continue the medications and monitor International Normalized Ratio (INR) to maintain it in the 2-3.

Case 2 

**Figure 2:**
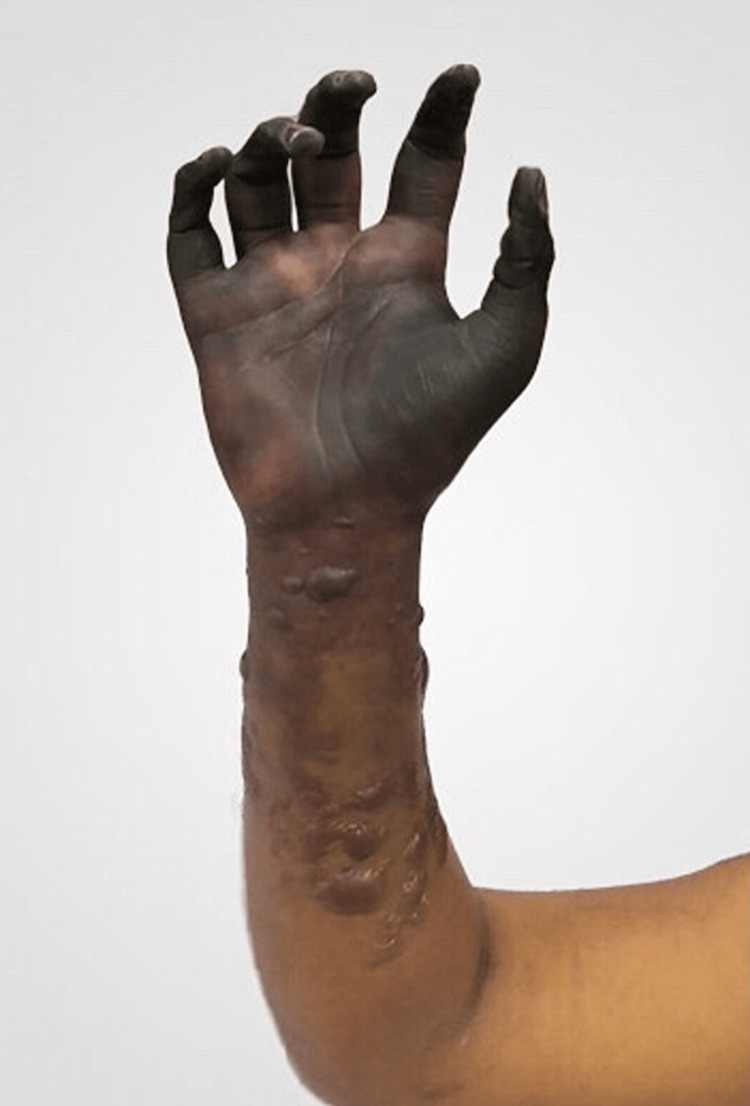
Dry gangrene of fingers and palm extending into the forearm. Multiple bullae on the forearm.

A 24-year-old primigravida, at 12 days postabortion, presented to the emergency department with gangrenous changes in her right hand and forearm. The patient was well 12 days ago when she got ultrasonography for two months of amenorrhea which was suggestive of 5+5 weeks of a blighted ovum. She got admitted to the hospital for pregnancy termination when an intra-arterial injection was injected in the brachial artery at the antecubital fossa, which resulted in bluish discoloration of her right hand and forearm. Spontaneous abortion took place, and ultrasonography of the pelvis showed no residual products of conception. The patient was put on broad-spectrum antibiotics. She was discharged home but soon complained of throbbing and intermittent shooting pain in the affected hand and forearm. On physical examination at 12 postabortion days, brachial artery pulsations were present, while radial artery pulsations were absent. Gangrenous changes had already set in the right hand and extended into the forearm (Figure 2). The patient was vitally stable, conscious, and oriented to time, place, and person. She was accepting oral feeds and passing stool and flatus. Ultrasound Doppler showed no flow and complete thrombus in the radial and ulnar artery's right mid and distal parts. CT Angiography could not visualize palmar and distal forearm vessels, while middle and proximal forearm vessels showed a heterogeneous appearance. Complete blood cell count was within normal limits, and liver function tests were mildly elevated on presentation, which gradually normalized. A forearm fasciotomy and debridement were done.

**Figure 3:**
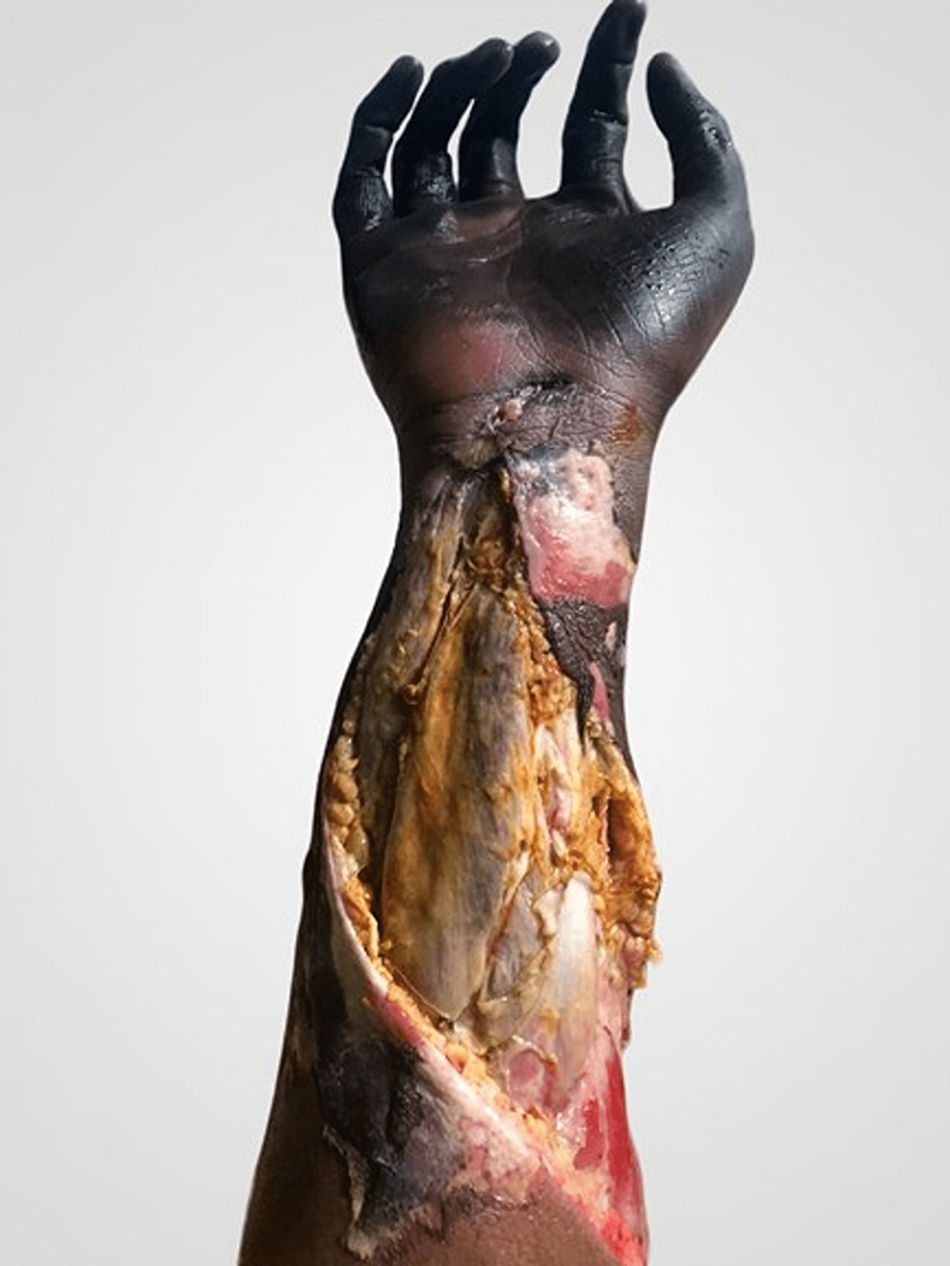
Post fasciotomy and debridement showing subcutaneous tissue, muscle, and tendons.

Intra-operatively opening the fascia, dead muscles popped out, then debrided (Figure 3). Amputation of the hand at the wrist had to be performed, which involved disarticulation at the wrist joint, followed by serial debridement and dressing. The patient was put on an aspirin tab 75 mg OD, IV piptaz 4.5 gm QID, and IV dalacin 600 mg TDS postoperatively. Tab cilostazol 100 mg BD was added to increase the blood flow. IV Heparin started during fasciotomy was discontinued after 48 hours, and Prothrombin Time and International Normalized Ratio (PT/INR) normalized. The fasciotomy incision was not closed and was left to heal by the second intention. The postoperative course of the patient was uneventful.

## Discussion

Epidemiology

Although brachial artery cannulation-induced severe ischemia is uncommon, post-cannulation gangrene has been researched and occasionally documented [6].

Pathogenesis and risk factors

The risk factors underlying this dreadful presentation are multifaceted and still under investigation. These include a combination of intrinsic patient factors that put the patient at risk of limb ischemia and external factors that exacerbate the risk.

Patient Factors

These include female gender, previous extremity injury or trauma, hypotension, hypercoagulable states, disseminated intravascular coagulation (DIC), and thrombotic thrombocytopenic purpura (TTP). The peripheral vascular disease has also been associated with postpartum gangrenes [7]. Systemic disorders are often associated with systemic gangrene, and sepsis is one of the most commonly associated systemic disorders [8]. Infection, especially infected emboli, could be a cause of ischemia. However, it was ruled out in our patients because ischemia was localized to the hand, there was no pus formation, and the pathology showed no evidence of infection [9]. Other conditions were also ruled out due to insufficient evidence to support the diagnoses. The physiological changes causing a hypercoagulable state during pregnancy can persist up to six weeks postpartum and significantly contribute to ALI in our patients [10].

Iatrogenic Factors

The use of Ergot alkaloid for pain management is also associated with postpartum gangrene [11]. Prior research has shown that a high dose of dopamine and noradrenaline is also associated with gangrene because of vasoconstriction [12]. The use of vasopressors is another risk factor. However, suppose the limb ischemia in our patients was due to vasopressor-related vascular spasm. In that case, there should also be evidence of ischemia in other body parts, which was not seen. The mechanism of drug-induced gangrene has not been specified but is believed to be accompanied by intravascular coagulation and DIC [13]. It can also be associated with intravenous cannulation, which may lead to unilateral gangrene. Increased cannulation time and early arterial injury are some of the risk factors [6]. Intraarterial cannulation is another etiological factor [6]. Case 2 reported a history of intraarterial cannulation, which, combined with her postpartum state, was likely the triggering factor for her ALI. Case 1 reported her symptoms to be triggered after intravenous cannulation, followed by significant swelling and ischemic changes in her hand. Mechanical injury to the brachial artery during iatrogenic cannulation attempts in the antecubital fossa exacerbates an already hypercoagulable state that is pregnancy and postpartum and is the most plausible cause of hand gangrene in our first case. 

Venous gangrene

Venous gangrene is a vital differential for patients presenting with symptoms suggestive of acute limb ischemia. It is a condition where the limb is swollen, and the veins are full due to venous occlusion. It can often be mistaken as acute limb ischemia since the edema can make it impossible to feel the pedal pulses. However, a Doppler scan will reveal normal distal waveforms and pressures [14]. In our patients, venous gangrene was initially considered as their medical history of recent pregnancy, and examination findings of limb swelling and suggestive skin changes supported the diagnosis. However: The clinical finding such as delayed capillary refill, cold fingers, and the ischemia pattern - starting from the right thumb and moving to the other digits and hand; The ultrasound Doppler showed decreased or no blood flow in the arteries; The pathological reports which showed arterial thrombosis, primarily through the radial side of the hand, all support that the problem was due to arterial thrombosis in our patients.

Unskilled healthcare workers' repeated cannulation attempts during intra-arterial or venous cannulation most likely caused intimal damage to the brachial artery, which triggered the coagulation cascade in the artery, resulting in thrombosis. Additionally, we believe the patient had some components of venous gangrene due to insufficient flow of arterial tree caused by thrombosis of the brachial artery.

Preventive measures

Healthcare worker education on taking extra precautions during cannulation of pregnant and post-pregnancy patients is needed. Patients should be made aware of this rare yet limb-threatening complication, especially those with a history of intraarterial cannulation during pregnancy or postpartum. The patient should be informed about the warning signs and symptoms of ensuing ischemia and the need for immediate medical consultation. Due to the high chances of developing ALI in postpartum patients, preventive measures should be ensured. Hands contain superficial and deep palmar arches for both arterial and venous supply. Through these arches, palmar digital arteries arise, which supply blood to digits. There is significant variability in the anatomy of these arches among people. Superficial arches are incomplete in 20% of the population, and incomplete arches can cause the diminished flow to the digits [15]. Thus, physical examination tests like Allen's should be done before performing radial artery cannulation in patients with ALI risk factors to assess these arches' competence and ensure adequate blood supply to the digits [16].

## Conclusions

The pregnancy and post-pregnancy phases are highly vulnerable due to the changing maternal physiology. Healthcare workers should be educated on the need for extra care during these patients' cannulation. The patients should be informed about the warning signs of ensuing ischemia and the need for immediate medical consultation if any are noticed. Patients with a history of intraarterial injection during or post-delivery should be provided extra care. Catastrophic limb-threatening conditions like acute limb ischemia and amputation can be prevented by quality improvement and health education of the staff and patients. Physical examination tests like Allen's test for the competence of the palmer arches should be incorporated in high-risk patients before cannulation to decrease the risk of this adverse outcome.
